# Modeling and experimental verification of polycaprolactone nanoparticle precipitation

**DOI:** 10.1038/s41598-026-35286-y

**Published:** 2026-01-29

**Authors:** Ewa Rybak, Jakub Trzciński, Jakub Gac, Tomasz Ciach

**Affiliations:** 1 Faculty of Chemical and Process Engineering, Warsaw University of Technology, Waryńskiego 1, Warsaw, 00-645 Poland; 2 Centre for Advanced Materials and Technologies CEZAMAT, Warsaw University of Technology, Poleczki 19, Warsaw, 02-822 Poland

**Keywords:** Polymeric nanoparticles, Nanoprecipitation, Polycaprolactone, Numerical modeling, Mathematics and computing, Nanoscience and technology

## Abstract

A numerical model based on the diffusion equation was developed to predict the size of polycaprolactone (PCL) nanoparticles produced via nanoprecipitation. The model requires minimal input data, making it cost-effective and experimentally efficient. It accounts for both diffusion-driven growth and the finite coalescence time of particles, a factor often overlooked in nanoparticle formation. Nanoparticles were synthesized under controlled variation of polymer concentration, surfactant amount, and mixing method, including microfluidics. The model demonstrated strong agreement with experimental data, yielding higher predictive accuracy than prior diffusion-limited models. It also enabled optimization of process parameters, improving control over size distribution and reducing aggregation. The proposed framework enhances nanoprecipitation scalability and reproducibility while lowering resource consumption. Its modular structure allows adaptation to other polymers and formulation conditions. This approach offers a practical and computationally efficient tool for the rational design of polymeric nanoparticles, with broad relevance to biomedical applications, including targeted drug delivery and nanomedicine.

**Supplementary Information:**

The online version contains supplementary material available at 10.1038/s41598-026-35286-y.

## Introduction

In recent years, polymeric nanoparticles (NPs) have been applied in drug delivery, diagnostics, and imaging^1–7^. It is assumed that they are nanoscale colloidal particles with sizes between 1 and 1000 nm^8^formed from polymers. This nanometric size equips NPs with a wide range of novel properties, e.g., optical, electronic, or magnetic^9,10^, without changing their chemical composition. The high surface-to-volume ratio and reduced molecular size of polymer chains^11^ contribute to enhanced material properties, such as lower viscosity and a lower glass transition temperature^12^.

Among various fabrication techniques, nanoprecipitation remains one of the most widely applied due to its simplicity, reproducibility, and scalability^13–16^. It involves mixing a polymer solution with a non-solvent, typically water, resulting in the spontaneous formation of nanoparticles without the need for high temperatures or pressures. Despite these advantages, precise control over particle size remains challenging due to the multivariable nature of the process, including polymer concentration, solvent ratio, surfactant content, and mixing dynamics. Moreover, the process often requires extensive experimentation and resources to achieve reproducible results^17^.

Microfluidic platforms offer improved control over nanoprecipitation by enabling rapid mixing at low Reynolds numbers, leading to more uniform particle populations^18–20^. However, they often involve complex device architectures and are not immune to the trial-and-error nature of formulation development. Regardless of the method, optimizing nanoparticle properties still demands time- and labor-intensive protocols. As a result, predictive tools are gaining interest in streamlining nanoparticle synthesis. Among numerical approaches, three main categories have emerged: molecular dynamics (MD), population balance models (PBM) coupled with computational fluid dynamics (CFD), and diffusion–coalescence models. MD simulations offer atomic-scale accuracy but are computationally intensive and primarily suited for mechanistic studies^21,22^. CFD–PBM models are robust tools for analyzing turbulent flow systems, but are resource-demanding and less applicable in laminar microfluidic contexts^23^. In contrast, models based on diffusion and particle coalescence equations provide a more accessible and practical alternative for simulating particle growth in systems dominated by Brownian motion and interfacial instability.

In this work, we develop a numerical model based on the diffusion equation with an added component for finite particle coalescence time, a parameter often overlooked in traditional approaches. This model requires only minimal input data and is computationally efficient. We validate it experimentally by making polycaprolactone (PCL) nanoparticles via nanoprecipitation, first comparing three mixing strategies (dropwise addition, one-shot mixing, and microfluidic flow focusing) and then applying the model to nanoprecipitation data obtained predominantly by one-shot and classical dropwise addition while systematically varying polymer concentration and surfactant amount. We aim to demonstrate that this streamlined modeling approach can accurately predict nanoparticle size and offer a versatile tool for optimizing formulation parameters in nanoparticle engineering.

## Model description

The model used in this work is based on the model proposed by Lebouille et al.^24^ The basic assumption of this model is to present the dynamics of nanoparticles coalescence as a second-order process. Thus, according to this model, the change in polymer nanoparticle concentration is described by the second-order equation:

1 $$\:\frac{d{c}_{NP}}{dt}=-K\bullet\:h\bullet\:{c}_{NP}^{2}$$

where $\:{c}_{NP}$ denotes the concentration of nanoparticles, and $\:h$ is the efficiency of collision ($\:h=1$ denotes the collision of two particles always leads to coalescence, $\:h=0$ – the coalescence never occurs). This value depends on the number of surfactant particles deposited at the nanoparticle’s surface.

The value of $\:K$ is the rate constant for particle-particle coalescence. In^24^, it is assumed that the collision of nanoparticles followed by coalescence is the result of diffusion only, which means this rate is expressed as:

2 $$\:K=4\pi\:D{\prime\:}R{\prime\:}$$

In above $\:D{\prime\:}$ denotes the sum of the diffusion coefficients of the reacting particles, and $\:R{\prime\:}$ is the sum of their radii (while colliding two particles with the same radii $\:R$ we have $\:{D}^{{\prime\:}}=2D$ and $\:{R}^{{\prime\:}}=2R$). The easiest way to computation of the diffusion coefficient is according to the Stokes-Einstein equation:

3 $$\:D=\frac{{k}_{B}T}{6\pi\:\mu\:R}$$

In the above equation $\:{k}_{B}$ denotes the Boltzmann constant, *T* – temperature of the process and $\:\mu\:$ – the viscosity of the liquid.

There are three quite important assumptions behind the description represented by the system of Eqs. (1–3):

- the particles retain a stable spherical shape - except for a short period of coalescence and formation of new, larger particles.
- the relative movement of the emerging polymer particles is the result only of diffusion (Brownian motion); coalescence caused by shear flow or sedimentation plays a negligible role here.
- very short (negligible) mixing time compared to the diffusion time - this means that after adding a PCL solution in an organic solvent to water, this solvent immediately mixes with water, and from the beginning, a system of isolated polymer particles in a poor solvent is considered.

The above assumptions may not be met for larger particles or particles with a non-spherical shape, such as fibrous particles^25^. In such a situation, the model should be modified accordingly. However, for the process considered in this article, the assumptions are met.

Such a set of conditions describes the so-called diffusion-limited coalescence (DLC)^26^, which is an analog of diffusion-limited aggregation (DLA), a widely used model of the formation of aggregates of solid particles.

This description should be considered sufficient for the dropwise case. In the case of a one-shot, it is necessary to consider the non-zero mixing time. In the case of microfluidics, it should be taken into account that, in addition to diffusion, the (laminar) flow field has a significant impact on the dynamics of particle collisions, which means that Eq. (2) should be replaced by a more detailed description of the particle movement in laminar flow.

In this work, in some simulations, the above model will be modified by taking into account a finite (non-zero) particle coalescence time. As it turns out, such a modification of the model allows for better agreement with the experiment, which means that in the described conditions, the coalescence time is important compared to the characteristic time of particle movement. The method of introducing a non-zero coalescence time and its impact on the numerically obtained results will be presented in the section ‘Influence of PCL concentration and the addition of co-solvent’.

This model essentially allows for determining the average diameter of the resulting particles. However, it can also be used to estimate the polydispersity (PDI) value. To do this, instead of considering a specific particle growth time, consider a range of values for that time, which yields a range of particle diameters, and based on that, a PDI value. This method is relatively imprecise (more accurate values can be obtained by knowing the exact distribution of particle growth times, which would require a more detailed analysis of the mixture’s hydrodynamics). However, as will be shown, it provides acceptable agreement with the experiment, at least in terms of the PDI trend.

## Results

### Mixing strategies and nanoparticle uniformity

**Fig. 1:**
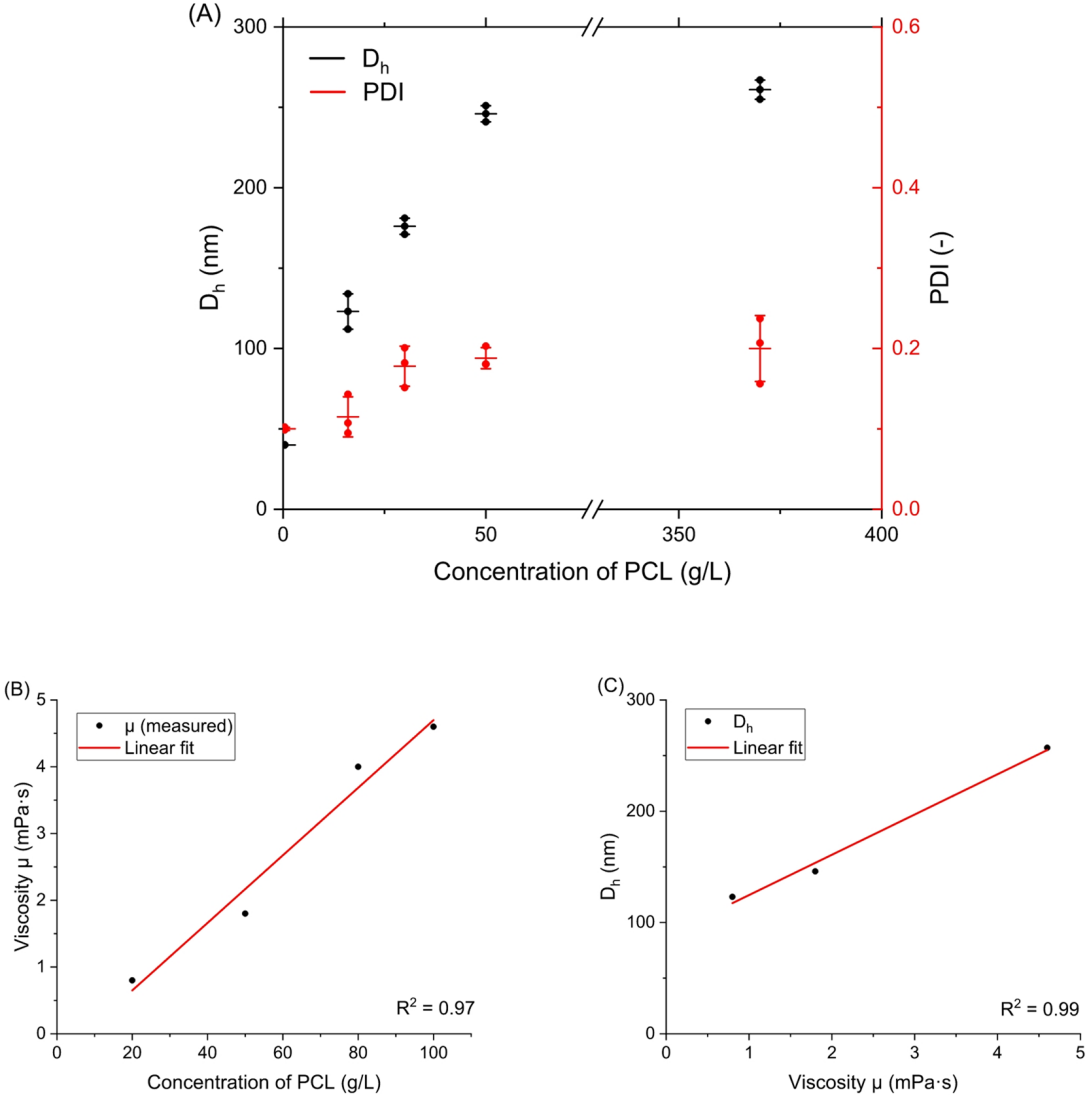
(**A**) DLS measurements of PCL nanoparticle suspensions obtained by one-shot nanoprecipitation with the addition of EtOH as a co-solvent, showing hydrodynamic diameter (D_h_) and polydispersity index (PDI) as a function of PCL concentration. Data are represented as individual points with mean ± standard deviation. (**B**) Apparent viscosity *µ* of PCL/THF solutions at 20 °C as a function of PCL concentration (20, 50, 80, and 100 g/L). Viscosity values were obtained by a rotational rheometer and averaged over the 20–80 s⁻¹ shear-rate plateau. The red line represents a linear fit (R² = 0.97), showing a monotonic increase of *µ* with polymer concentration. (**C**) Correlation between *µ* of the organic phase and the hydrodynamic diameter D_h_ of PCL nanoparticles obtained by one-shot nanoprecipitation in THF/EtOH at the PCL concentrations for which reliable DLS data were available (20, 50, and 100 g/L). A linear fit (R² = 0.99) indicates a strong positive correlation between organic-phase viscosity and final nanoparticle size, supporting the proposed viscosity-controlled coalescence mechanism.

**Fig. 2:**
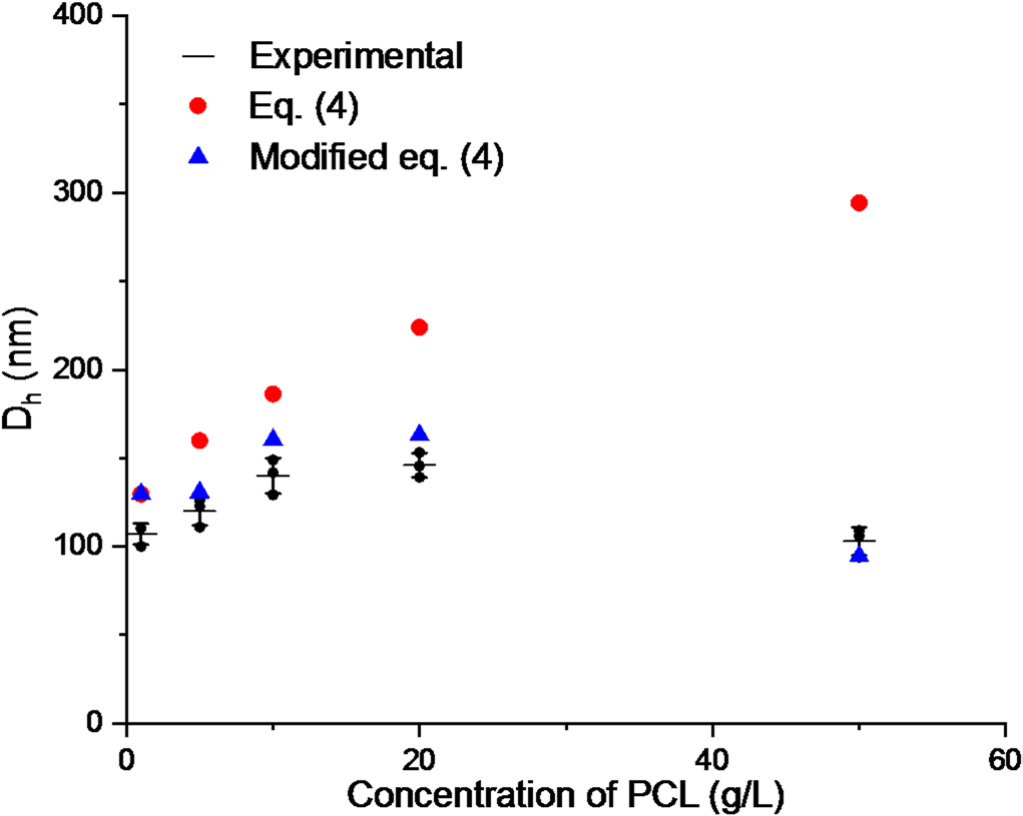
Hydrodynamic diameter of PCL nanoparticles as a function of polymer concentration. Experimental data for nanoparticles prepared by the dropwise method (black dots, mean ± SD, *n* = 3, DLS) compared with numerical predictions obtained from Eq. (4) (red circles) and from the modified Eq. (4) including a non-zero coalescence time (blue triangles).

**Fig. 3:**
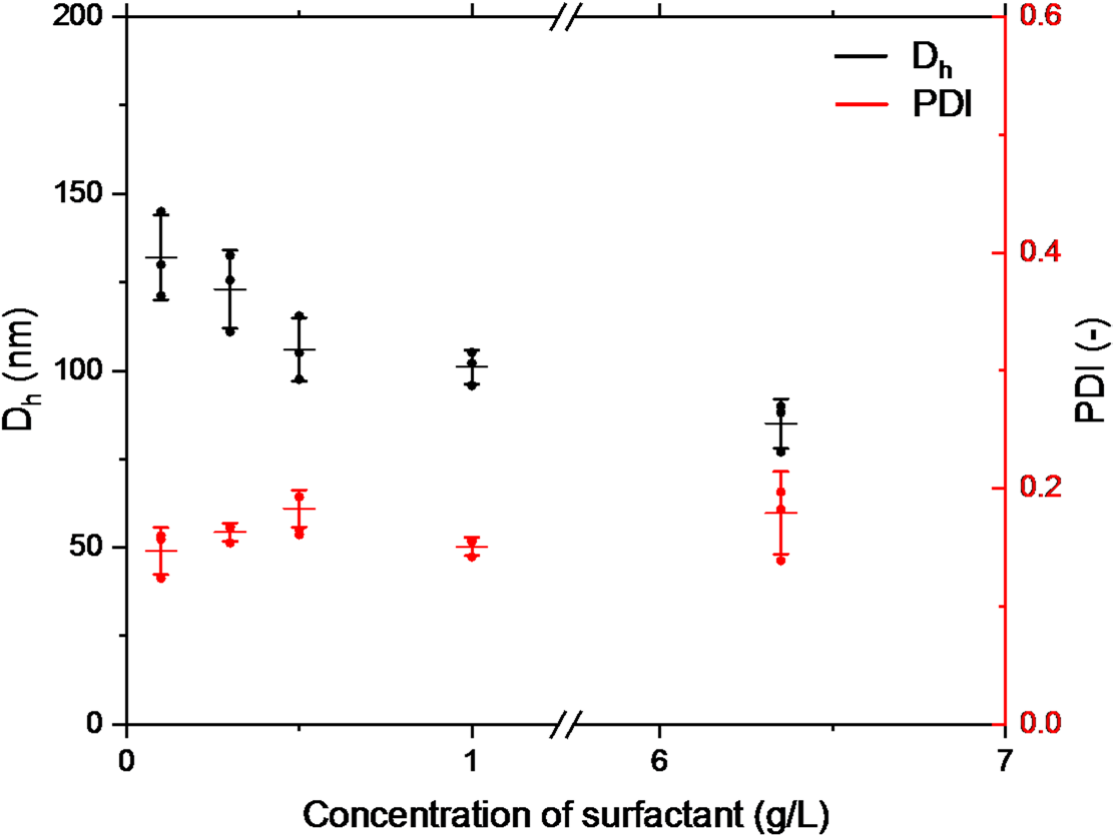
DLS measurements of samples formulated by the dropwise method with different surfactant concentrations (*n* = 3). Data are represented as individual points with mean ± standard deviation.

**Fig. 4:**
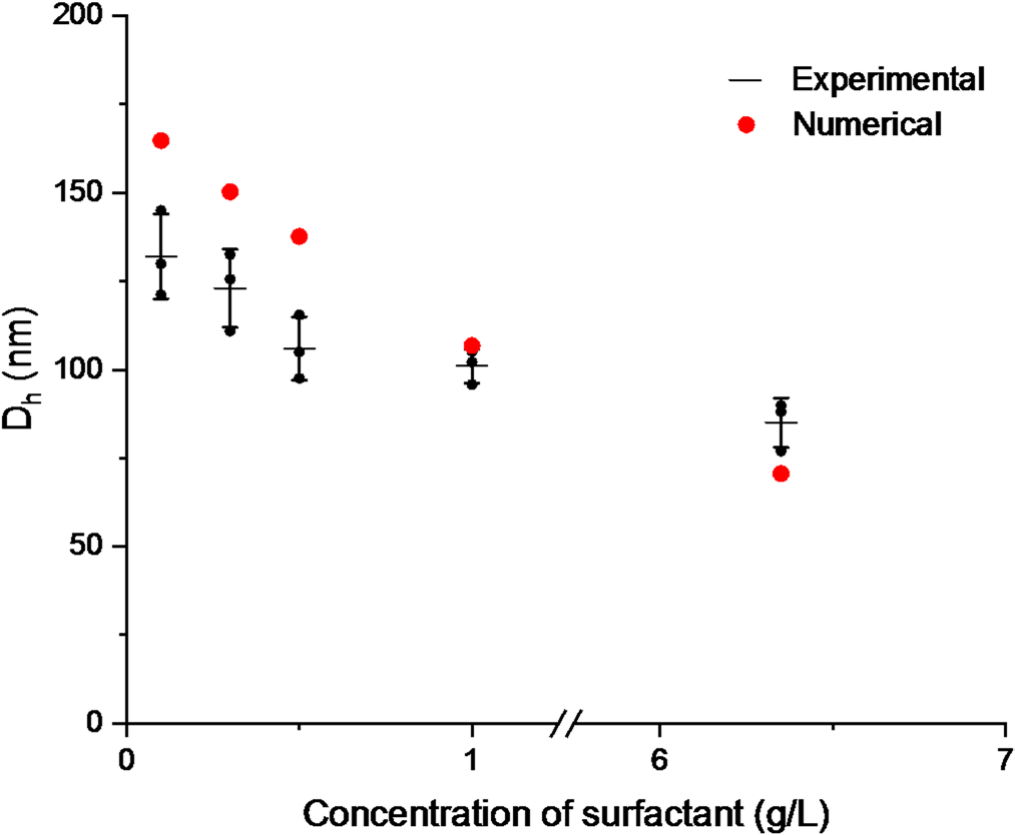
Experimental hydrodynamic diameter D_h_ of PCL nanoparticles prepared by the dropwise method (black dots, mean ± SD, *n* = 3, DLS) compared with numerical predictions (red dots) as a function of surfactant concentration.

**Fig. 5:**
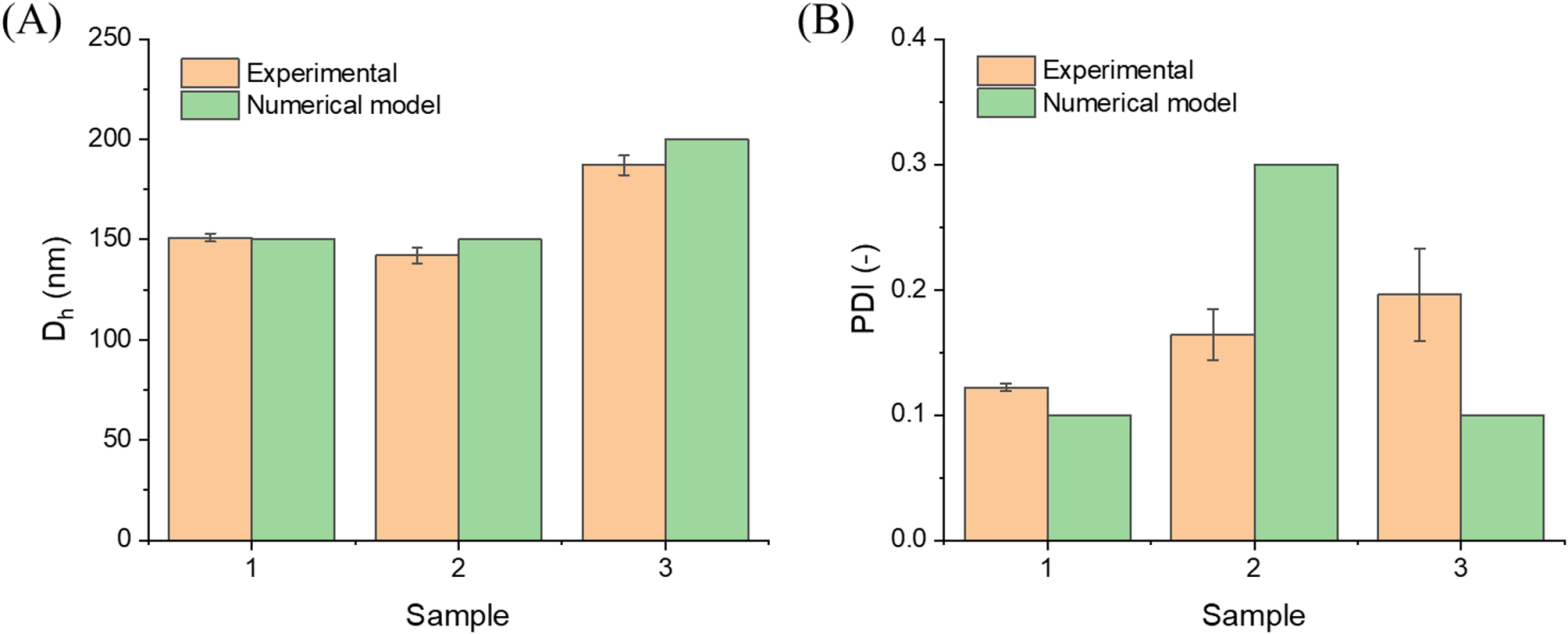
Comparison between the target (assumed) and experimental values of (**A**) hydrodynamic diameter (D_h_) and (**B**) PDI for three designed PCL nanoparticle formulations obtained using the one-shot method (*n* = 3).

To evaluate the impact of mixing technique on nanoparticle characteristics, three formulation strategies were compared: dropwise addition, one-shot mixing, and microfluidic flow focusing. Under the optimized, pre-validated conditions used in this study, all three methods produced PCL nanoparticles of similar average size across the tested concentrations. A two-way ANOVA with the nanoprecipitation method and PCL concentration as fixed factors confirmed that the main effect of mixing strategy on hydrodynamic diameter (D_h_) and PDI was not statistically significant. In contrast, PCL concentration significantly affected D_h_ (*p* = 0.0011). These findings indicate that, under the conditions investigated in this study, the choice of mixing strategy does not measurably influence mean particle size or polydispersity. This observation should therefore be interpreted within the context of controlled experimental protocols, rather than as a general rule applicable to all possible parameter ranges. A full comparative analysis of particle size and PDI distributions across methods is available in Supplementary Note 1 and Supplementary Figure S2. Based on these results, we used different nanoprecipitation protocols for different parts of the study. PCL nanoparticles in Fig. 1 (co-solvent addition) were prepared using the one-shot method. In contrast, all surfactant-screening experiments and PCL concentration studies in Figs. 2, 3 and 4 were carried out using the classical dropwise nanoprecipitation protocol. Figure 5 (model verification) again presents data obtained with the one-shot method, using the same organic phase composition as in the co-solvent one-shot series (Fig. 1). Microfluidic flow-focusing was employed only for the comparative study summarized in Supplementary Note 1 and Supplementary Figure S2. On this basis, the subsequent modeling and design studies focused on the representative dropwise and one-shot datasets, while microfluidic data were retained in the Supplementary Information to document that the three mixing strategies yield comparable nanoparticle sizes under our conditions.

### Influence of PCL concentration and the addition of co-solvent

The NP size and growth are controlled by the solvent composition during the preparation process^27,28^. To improve the robustness and reproducibility of one-shot nanoprecipitation across the investigated PCL concentration range, ethanol (EtOH) was added to tetrahydrofuran (THF) as a water-miscible co-solvent (EtOH: THF = 1:2, v/v). Among the three tested EtOH: THF ratios (1:1, 1:2, 2:1, v/v), the 1:2 ratio yielded the most favorable particle size and PDI. Detailed Dynamic Light Scattering (DLS) data for each solvent ratio are provided in Supplementary Tables S1 and S2. Because both THF and EtOH are fully miscible with water, EtOH primarily modifies solvent composition (polarity/solvent quality) and the solvent-exchange pathway upon injection, improving reproducibility and colloidal stability in the one-shot protocol.

Figure 1 summarizes the experimental DLS data for PCL nanoparticles obtained by one-shot nanoprecipitation with EtOH as a co-solvent, showing how both D_h_ and PDI evolve with increasing polymer concentration. These measurements provide the experimental trend used for subsequent model fitting and validation, while the highest concentrations mainly illustrate the behavior in the upper range explored in this study. With increasing PCL concentration, D_h_ and PDI increased (Fig. 1A). This may be addressed by increasing the initial polymer concentration, which promotes particle growth more than nucleation, thereby forming larger particles^29^.

To provide quantitative support for this viscosity-based mechanism, we measured the viscosity of model PCL solutions in THF (without EtOH co-solvent) at PCL concentrations of 20, 50, 80, and 100 g/L at 20 °C using a rotational rheometer (Fig. 1B). For reference, the viscosity of the neat EtOH: THF mixture (1:2, v/v) at 20 °C was approximately 0.6 mPa·s, i.e., close to that of pure THF (0.5 mPa·s), indicating a negligible effect of ethanol on the baseline organic-phase viscosity at the fixed solvent ratio used here. Therefore, we treated the viscosity trend measured for PCL/THF as a practical proxy for the corresponding PCL solutions in the THF/EtOH one-shot series, because the polymer contribution dominates the overall viscosity increase. The apparent viscosity of the organic phase increased from approximately 0.8 to 4.6 mPa·s over this concentration range. Because reproducible nanoparticle suspensions were available only for 20, 50, and 100 g/L PCL in the one-shot series, the correlation with D_h_ was constructed for these three concentrations. Even within this subset, a strong positive linear relationship was observed between viscosity and D_h_ (Fig. 1C, R² = 0.99), confirming that larger particle sizes are associated with a more viscous organic phase.

At higher PCL concentrations, the organic-phase viscosity increases and the system may approach or exceed the overlap concentration c*, which can hinder early-stage mixing and reduce nanoprecipitation control (promoting coalescence and broader size distributions). For our PCL (M_w_ = 14,000 g/mol) in THF, $\:{c}^{*}$can be estimated as $\:{c}^{*}\sim\:1/\left[\eta\:\right]$, giving c*≈ 43 g/L based on literature Mark–Houwink data at 25 °C^30^. In this context, the very high-concentration one-shot series with EtOH assistance (up to 370 g/L) is reported as the highest concentration range explored in this study. In contrast, the EtOH-free dataset considered in the subsequent analysis was limited to lower concentrations (up to 50 g/L), as higher concentrations without EtOH did not yield reproducibly stable nanoparticle suspensions. A more detailed, model-based interpretation of the concentration dependence is presented later in the manuscript.

The influence of PCL concentration on particle diameter may also be described using the numerical model presented above. Let us start with the system with EtOH as a co-solvent. Using a fully water-miscible THF/EtOH organic phase supports the assumption of rapid solvent exchange adopted in the numerical description; consequently, after the initial mixing step, particle motion is treated as diffusion in a water-rich continuous phase, and *µ* in Eq. (3) is approximated by the viscosity of water at 300 K (Table 1).

The Eqs. (1–3) may be transformed to the equation describing the evolution of particle diameter on time $\:{D}_{h}\left(t\right)$ in form:

4 $$\:\frac{d{D}_{h}}{dt}=\frac{8}{3}\frac{{k}_{B}T}{\mu\:}\frac{{c}_{p0}{D}_{h,0}^{3}}{{D}_{h}^{2}}$$

where $\:{D}_{h,0}$ is the initial value of particle diameter. The solution of (4) is:

5 $$\:{D}_{h}\left(t\right)={D}_{h,0}{\left(1+\frac{t}{{\tau\:}_{cs}}\right)}^{\raisebox{1ex}{$1$}\!\left/\:\!\raisebox{-1ex}{$3$}\right.}$$

where $\:{\tau\:}_{cs}=\frac{3}{8}\frac{\mu\:}{{c}_{p0}{k}_{B}T}$ is the time scale of coalescence. Equation (5) allows us to compute the final value of particle diameter when we know the values of the initial particle diameter $\:{D}_{h,0}$, time of a process *t* and the initial number concentration of particles per volume unit $\:{c}_{p0}$. The last quantity is connected with the initial particle diameter and the mass concentration of PCL $\:{c}_{0,mass}$ by means of the formula:

6 $$\:{c}_{p0}=\frac{{c}_{0,mass}}{{m}_{p}}=\frac{6{c}_{0,mass}}{\pi\:{D}_{h,0}^{3}{\rho\:}_{PCL}}$$

Merging (5) and (6) together, we obtain:

7 $$\:{D}_{h}\left(t\right)={D}_{h,0}{\left(1+16\frac{{k}_{B}T}{\mu\:}\frac{{c}_{0,mass}}{\pi\:{\rho\:}_{PCL}}\frac{t}{{D}_{h,0}^{3}}\right)}^{\raisebox{1ex}{$1$}\!\left/\:\!\raisebox{-1ex}{$3$}\right.}$$

In the above equation, all the quantities are constant apart from $\:{c}_{0,mass}$ (which is set for the experiments), and the time of particle formation *t* and their mean initial diameter $\:{D}_{h,0}$. The last ones, however, may be found by fitting the solution of (7) to the experimental results. The diameter determined from both the model equations and DLS measurements is expressed as a number-average value. In this context, PDI values ≤ 0.2 are generally considered acceptable for polymer-based nanoparticles^31–34^, and under such conditions, the difference between number- and Z-average sizes remains modest (< 20%)^33^. E.g., the results of the system with ethanol addition, the best fitting is obtained for the parameter values as given in Table 1.

**Table 1:** Constant and fitted parameters for the particle growth model.

Constant parameters				Fitted parameters	
$$\:{k}_{B}$$, J/K	$$\:T$$, K	$$\:\mu\:$$, mPa·s ^*^	$$\:{\rho\:}_{PCL}$$, $$\:\raisebox{1ex}{$g$}\!\left/\:\!\raisebox{-1ex}{$c{m}^{3}$}\right.$$	$$\:{D}_{h,0}$$, nm	$$\:t$$, ms
1.380649 × 10^− 23^	300	0.9321	1.145	41.0	16.0

^*^Assumed as the viscosity of water at a temperature of 300 K.

Adding EtOH as a co-solvent proved beneficial for stabilizing nanoparticle suspensions. This aligns with findings in the literature^35^, which suggest that co-solvents enhance uniform solvent diffusion, resulting in more homogeneous particle distributions. However, our results further reveal the potential drawbacks of EtOH, such as increased particle size at higher concentrations, providing a balanced view of its role in nanoprecipitation processes.

In the absence of co-solvent (EtOH), the formula (7) is still valid for the relatively low (up to 10 g/L) concentration of PCL. The difference between the calculated and measured particle diameter values ​​increases for greater values. In particular, the latter decreases with increasing PCL concentration, which is not predicted by Eq. (7).

To describe this effect, Lebouille et al.^24^ proposed the modification of (4) by adding the factor *h* on the right-hand side of this formula:

8 $$\:\frac{d{D}_{h}}{dt}=\frac{8}{3}\frac{{k}_{B}T}{\mu\:}\frac{{c}_{p0}{D}_{h,0}^{3}}{{D}_{h}^{2}}h\left(x\right)$$

Where $\:x$ is defined as a fraction of the particle surface “blocked” by the surfactant molecules. It arises that when $\:x=0$ (no surfactant molecules at the surface of PCL particles), we have $\:h\left(x\right)=1$, and (4) is still valid. In contrast, when $\:x=1$ (the surfactant blocks the surface), no coalescence is possible ($\:h\left(x\right)=0$). Beyond these extreme cases, the value of $\:h\left(x\right)$ remains unknown. However, one may expect that the diffusion of surfactant particles is much faster than the diffusion of PCL particles; thus, the final fraction *x* is reached at a relatively short time compared to the time of particle growth. The surfactant at the surface of particles increases the effective surface tension coefficient, thus slowing down the coalescence process alone. In fact, (4) predicts that when two particles meet, they immediately form the final spherical particle with a volume equal to the sum of the volumes of both particles. When the surfactant partially blocks the surface of particles, the act of coalescence is no longer instantaneous. The above experimental results, in comparison with the results predicted by the model with an instantaneous coalescence time, show that this issue requires a more detailed description. To take into account the non-zero coalescence time, it should therefore be assumed that before the two merging particles achieve the final shape of a sphere with a volume equal to the sum of the volumes of both particles, they will have a “transitional” shape for some time. This transitional shape will initially be close to two tangent spheres, and will then resemble an ellipsoid, with the ratio of the semi-major to minor axis gradually approaching unity. However, before this happens, the particle formed as a result of coalescence will be elongated in one direction, which will increase the probability of its collisions with other particles.

A detailed quantitative description of the changes in the shape of a particle/droplet resulting from the coalescence of two smaller particles was presented in the works by Garabedian and Helble^36,37^. According to their description, the two coalescing droplets take the shape described in polar coordinates $\:r,\vartheta\:$ by the equation:

9 $$\:{r}^{2}\left(\vartheta\:\right)={D}_{h,0}^{2}\bullet\:b\bullet\:c\left(b\right)\bullet\:\left(1-b\bullet\:{sin}^{2}\left(\vartheta\:\right)\right)$$

Where *b* is parameter of the shape ($$\:b\to\:1$$ denotes two separate touching spheres, $$\:b=0$$ – one larger sphere) and $$\:c\left(b\right)$$ is a parameter ensuring the preservation of the volume of merging particles (more details in^36^). The “neck” thickness of coalescing spheres is obtained for $$\:\vartheta\:=\raisebox{1ex}{$\pi\:$}\!\left/\:\!\raisebox{-1ex}{$2$}\right.$$ and is equal to:

10 $$\:L={D}_{h,0}\sqrt{b\bullet\:c\left(b\right)\bullet\:\left(1-b\right)}$$

and is dependent on the nondimensional time of process^36^:

11 $$\:\frac{L}{{D}_{h,0}}=\mathrm{m}\mathrm{i}\mathrm{n}(1,\:0.1436*ln\tau\:+0.5347)$$

Where nondimensional time $\:\tau\:$, the time between two successive collisions, is proportional to the inverse of the collision frequency $\:f$. This, in turn, is proportional to the square of PCL concentration because the average number of collisions between particles is proportional to the square of the number of particles (when we consider only the most important double collisions):

12 $$\:\tau\:\propto\:\frac{1}{f}\propto\:\frac{1}{{c}_{p0}^{2}}$$

The proportionality factor depends on the surfactant concentration, particle size, and the diffusivity of the surfactant. In this case, the maximum mean particle diameter for a given PCL concentration, approximately 20 g/L, can be chosen. The final particle diameter is computed from (7) and corrected by factor (11). The results are presented in Fig. 2.

### Influence of surfactant concentration

The presence of surfactant has a strong influence on nanoparticle formation. For each PCL concentration investigated, formulations containing Pluronic^®^ F-127 (F127, 5 g/L) consistently exhibited higher hydrodynamic diameters and lower PDI values than surfactant-free samples, confirming that steric stabilization by adsorbed F127 efficiently suppresses aggregation driven by polymer–polymer interactions during precipitation^38^. Building on this observation, we next systematically varied the F127 concentration (Fig. 3) to determine the minimum surfactant level required to achieve stable, narrowly distributed PCL nanoparticle suspensions.

Figure 3 shows the effect of surfactant concentration on the size and distribution of the formulated NPs. In these experiments, the PCL concentration in the organic phase was kept constant at 50 g/L; only the F127 concentration in the aqueous phase was varied. Increasing F127 from 0.10 to 0.50 g/L reduced the hydrodynamic diameter from 132 ± 12 nm to 106 ± 9 nm, while PDI increased slightly from 0.147 ± 0.020 to 0.183 ± 0.016. At low F127 levels, surfactant molecules may not adsorb quickly enough to stabilize newly formed particles, leading to aggregation due to hydrophobic interactions^39^. In contrast, higher concentrations lower surface tension and better stabilize particles, resulting in smaller sizes^40^. If stabilization is insufficient, aggregation and instability may occur^41^. At higher F127 concentrations (≥ 1 g/L), the trend remains consistent, with D_h_ decreasing further and PDI showing a tendency to increase, indicating more heterogeneous size distributions at the highest surfactant levels.

To analyze the system with surfactant, Eq. (4) should be fulfilled with the coefficient *h*, which denotes the degree of surface blocking with surfactant^24^. Thus, the equation would take the form:

13 $$\:\frac{d{D}_{h}}{dt}=\frac{8}{3}\frac{{k}_{B}T}{\mu\:}\frac{{c}_{p0}{D}_{h,0}^{3}}{{D}_{h}^{2}}h$$

The coefficient *h* generally depends on the concentration of surfactant molecules attached to the droplet surface. It is obvious that in the absence of surfactant – and also at the onset of the process when there are no surfactant molecules attached to the particle surface, is equal to zero – we should set $\:h=1$. Further on, when surfactant molecules appear on the surface, the value of *h* decreases and finally reaches 0 when the whole surface is blocked by the surfactant.

The exact form of the dependence $\:h\left({c}_{s,att}\right)$, where $\:{c}_{s,att}$ denotes the concentration of surfactant molecules attached to the surface of particles – is not known^24^. However, one may assume that this coefficient will be proportional to the quotient of the surface of particles covered with a surfactant to the total surface of particles. The total surface of particles is given by the equation:

14 $$\:A={c}_{p}\bullet\:\frac{\pi\:{D}_{h}^{2}}{4}$$

Where $\:{c}_{p}$ is the number concentration of particles; from mass conservation law, we have $\:{c}_{p}={c}_{p,0}\frac{{D}_{h,0}^{3}}{{D}_{h}^{3}}$.

The surface “blocked” by the surfactant may be expressed in the form:

15 $$\:{A}_{block}={c}_{s,att}\frac{\pi\:{a}^{2}}{4}$$

In above, $\:{c}_{s,att}$ denotes the concentration of surfactant molecules attached to the particle’s surface, and $\:a$ – the equivalent diameter of the surfactant molecule. It may differ from the actual diameter of the molecule (and it is expected to be greater than the real diameter) because the molecules do not arrange themselves at the interface in a way that ensures the tightest packing.

Thus, the value of coefficient *h* is given as:

16 $$\:h=\left\{\begin{array}{c}1-\frac{{A}_{block}}{A}\:for\:{A}_{block}<A\:\\\:0\:otherwise\end{array}\right.$$

The Eq. (13) should also be completed with the equation describing the diffusion of surfactant to the particle surface. This equation takes the form:

17 $$\:\frac{d{c}_{s,att}}{dt}=\frac{2}{3}\frac{{k}_{B}T}{\mu\:}\frac{{D}_{h}}{{D}_{s}}\left({c}_{s,0}-{c}_{s,att}\right){c}_{p}h$$

In the above equation, $\:{D}_{s}$ denotes the equivalent diameter of the surfactant molecule to diffusion, which may – or may not – be equal to $\:a$ and $\:{c}_{s,0}$ – initial concentration of surfactant.

The parallel solution of Eqs. (13) and (17) with conditions (14–16) yields the results presented in Fig. 4.

Both the simulations and experimental results demonstrate a consistent trend: particle diameter decreases with increasing surfactant concentration at low concentrations, while at higher surfactant levels, further size reduction becomes modest as interfacial adsorption approaches saturation. In addition, micellization at sufficiently high concentrations can reduce the fraction of free (non-micellar) surfactant available for adsorption, further attenuating the effect of additional bulk surfactant. To provide a clearer overview of the experimental findings, the effects of formulation and process parameters on nanoparticle size and distribution are summarized in Supplementary Table S3, which complements the detailed discussion presented in the preceding subsections.

The obtained PCL nanoparticles exhibited zeta potentials ranging from − 11 to − 15 mV. Although below the ± 30 mV threshold typically associated with strong electrostatic stabilization, these values do not imply instability. Importantly, steric stabilization by Pluronic^®^ F-127 compensates for the lower zeta potential, preventing aggregation. In our dispersions, the combination of a negative surface charge and a hydrated F127 corona effectively suppresses close particle–particle contacts and reduces the probability of irreversible coalescence, which is consistent with the absence of visible sedimentation or changes in D_h_ and PDI over the measurement timescale. This behaviour aligns with previous reports on polymeric nanoparticle systems stabilized by non-ionic surfactants, where moderate negative zeta potential values (on the order of − 10 to − 20 mV) are sufficient to ensure colloidal stability when steric repulsion is present. Such combined steric and electrostatic effects are commonly enough to maintain nanoparticle dispersion in systems stabilized with non-ionic surfactants^42–44^.

### Model verification

In this step, our goal was not to refit the model parameters, but to verify whether the model can be used in a “design” mode to identify formulation conditions leading to a desired nanoparticle size distribution. To this end, we predefined three target nanoparticle populations (Table 2). Using the previously established relationships between PCL concentration, surfactant amount, and the resulting D_h_ and PDI, the model was then used inversely to predict the polymer and surfactant concentrations required to obtain each of these targets. The corresponding PCL NP suspensions were subsequently prepared in the laboratory using the one-shot method and characterized by DLS, as shown in Table 2; Fig. 5.

**Table 2:** Summary of the design-mode use of the model: target nanoparticle populations, model-predicted formulation conditions, and experimental results with relative errors (one-shot method, *n* = 3).

Formulation	Target		Predicted		Experimental		Relative error	
	D_h_ (nm)	PDI (-)	PCL (g/L THF)	F127 (g/L H_2_O)	D_h_ (nm)*	PDI (-)*	D_h_ (%)†	PDI (%)†
1	150	0.1	5.5	4	151 ± 2	0.122 ± 0.003	1	22
2	150	0.3	30	6	142 ± 4	0.164 ± 0.020	5	45
3	200	0.1	9.5	0,5	187 ± 5	0.196 ± 0.037	7	96

*Mean ± standard deviation, *n* = 3.

†Relative error calculated as $\:\mid\:\mathrm{experimental\:mean}-\mathrm{target}\mid\:/\mathrm{target}\times\:100\mathrm{\%}$

Overall, the experimental results validated the model’s ability to predict formulation conditions, particularly with respect to the diameter of the resulting nanoparticles. Across the three designed formulations, the relative differences between the target and experimentally measured diameters ranged from 1% to 7%, which is comparable to the experimental variability typically reported for DLS-based size measurements of polymer nanoparticles prepared by nanoprecipitation and, therefore, considered acceptable for such systems^45,46^. In contrast, the deviations in PDI were larger (22–96%). This discrepancy is consistent with the higher experimental uncertainty of PDI, which is intrinsically sensitive to small changes in aggregation, colloidal stability, and measurement protocol in DLS. Following ISO 22412:2025, cumulants analysis relies on the Taylor series expansion of the autocorrelation function, where the first-order term yields the z-average size, and the second-order term provides the PDI as a measure of distribution width; the standard ensures comparability across DLS instruments. It is also worth noting that the PDI targets used in the design step should be viewed as approximate guidelines rather than exact values. For formulation 2, for example, a target PDI of 0.30 was chosen to represent a more polydisperse case; based on our previous experiments, the actual PDI expected for these formulation conditions is somewhat lower (closer to 0.20), but still clearly higher than in formulation 1. The fact that the measured PDI lies in this range, while exceeding that of formulation 1, indicates that the model is more reliable in capturing relative trends in polydispersity than in its exact magnitude. Nevertheless, this level of agreement is sufficient for using the model as a practical process design tool, with D_h_ being targeted quantitatively and PDI being interpreted in a relative sense to distinguish narrower versus broader size distributions.

Table 3 further contextualizes the proposed model’s performance by comparing its accuracy, computational efficiency, and scalability with other widely used modeling approaches, such as Molecular Dynamics (MD) and Population Balance Models (PBM).

**Table 3:** Comparison of modeling approaches.

Model type	Application domain	Validation against experiments	Computation time (relative)	Scalability
MD^22,47^	Surface chemistry, nucleation, coating systems	atomic-level accuracy for interfacial processes; not directly predictive of particle size distribution	High	Low
PBM^48,49^	Flow dynamics, turbulent systems, nanoprecipitation	good agreement with particle size distribution and droplet dispersion	Medium	Medium
Our model	Nanoprecipitation of polymeric nanoparticles	high accuracy in number-average size prediction	Low	High

MD offers very high accuracy at the atomistic level and provides valuable mechanistic insights. Still, it is not suitable for predicting particle-size distributions and remains computationally too demanding for large-scale or real-time use. PBM has been successfully applied to turbulent multiphase systems and, more recently, to nanoprecipitation of lipid nanoparticles, showing good agreement with experimental PSDs. However, its scalability is limited, especially when coupled with detailed flow simulations. In contrast, our model combines high predictive accuracy with low computational cost, making it particularly well-suited for nanoprecipitation, where fast and scalable predictions of nanoparticle size are essential. This comparison underscores the practical advantages of our approach for process optimization.

## Discussion

Increasing polymer concentration raises the viscosity of the organic phase, leading to larger droplets and, consequently, larger nanoparticles^50^. The observed relationship between polymer concentration and droplet size emphasizes the importance of controlling viscosity to achieve precise nanoparticle properties, particularly in scaling up production for pharmaceutical applications. At the highest polymer concentrations, the system may also approach or exceed the chain overlap concentration c*, allowing chain overlap/entanglement effects to further slow down early-stage mixing and amplify coalescence-driven broadening of the size distribution. This is consistent with the c* estimate discussed above for PCL in THF. In contrast, higher concentrations of Pluronic^®^ F-127 reduce particle size by preventing droplet coalescence through interfacial adsorption^51–53^.

Overall, the three mixing approaches investigated here—dropwise addition, one-shot addition, and microfluidic mixing—yielded similar particle diameters, with the dominant trends governed by PCL and surfactant concentration. Consistent with the two-way ANOVA results showing no significant main effect of mixing strategy on either D_h_ or PDI (*p* > 0.05), the model was developed and validated using representative datasets obtained with the dropwise and one-shot protocols. Adding EtOH to the organic phase changes the solvent composition and can shift the balance between nucleation and growth during solvent exchange, which in our experiments was reflected by larger D_h_ (and, at the upper concentrations, higher PDI)^35^. This indicates that EtOH functions primarily as a processing aid in one-shot nanoprecipitation, improving the robustness and stability of the obtained suspensions rather than minimizing particle size.

The proposed numerical model, based on the diffusion equation, predicts particle diameter at low PCL concentrations with good agreement. At higher concentrations, incorporating non-zero coalescence time accounts for particle-particle interactions and non-spherical shapes, improving prediction accuracy. Unlike CFD-population balance models by Cheng and Fox^23^, our approach is computationally efficient and better suited for laminar microfluidic systems. A key innovation is the inclusion of surfactant-blocking dynamics, which enables the realistic simulation of colloidal stabilization and provides a practical tool for designing nanoparticles with tailored interfaces for drug delivery and imaging applications.

Our numerical model shows strong agreement with experimental data, offering improved predictive accuracy for nanoparticle size compared to earlier diffusion-limited approaches, which provided valuable mechanistic insights but typically reported only qualitative agreement with experiments^24,26^. Incorporating surfactant surface-blocking dynamics improved accuracy in high-surfactant systems, addressing limitations of earlier models^54,55^. Some discrepancy stems from simplifications such as ideal mixing, uniform particle distribution, and constant interaction time. These assumptions overlook spatial variations, local concentration gradients, and multi-particle interactions, which may explain differences in PDI, though they have minimal impact on average particle size.

## Conclusions

In this work, we developed and experimentally validated a diffusion-based numerical model for predicting the size of polycaprolactone nanoparticles formed by nanoprecipitation. By systematically varying polymer concentration, solvent composition, and surfactant content, and by comparing three mixing strategies (dropwise, one-shot, and microfluidic), we demonstrated that diffusion-limited coalescence with finite coalescence time and surfactant surface blocking can quantitatively capture the dependence of nanoparticle size on formulation and process parameters. By introducing a finite coalescence time and surfactant surface-blocking into a diffusion-limited coalescence framework, the model achieved good quantitative agreement with experimentally measured number-average diameters and qualitatively captured trends in PDI. The computationally efficient approach provides a practical design tool for optimizing nanoprecipitation-based polymeric nanoparticle formulations for biomedical and industrial applications. Overall, it provides a robust tool for nanoparticle design, contributing to colloid science by bridging the gap between simulation and experiment in the development of stable, scalable colloidal systems.

## Methods

### Materials

Polymer, ε -Polycaprolactone (PCL) with a weight average molar mass of 14,000 g/mol, Pluronic^®^F-127 (F127), were purchased from Sigma-Aldrich/Merck (Poznań, Poland), and tetrahydrofuran (THF) (HPLC grade, purity 99.9%) was obtained from Chempur (Piekary Śląskie, Poland). Ethanol (EtOH) was purchased from Stanlab (Lublin, Poland). The antisolvent phase was ultrapure water (conductivity = 13.4 µS/cm) produced by reverse osmosis (Milli-Q^®^, Millipore, Burlington, MA, USA).

### Methods of NP formulation

**Fig. 6:**
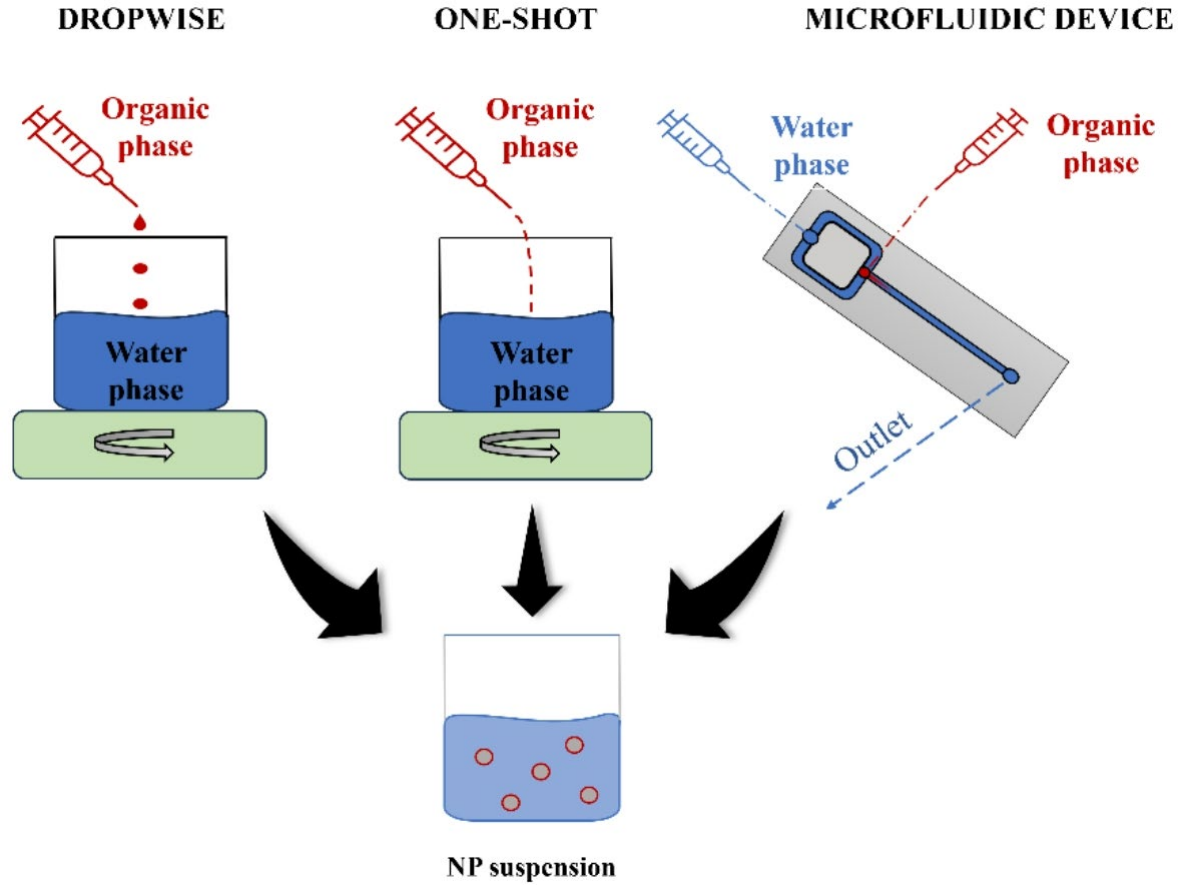
The methods used for NP formulation.

PCL nanoparticles were prepared using a two-step method. Firstly, the polymer was dissolved in a solvent and added to a non-solvent in three ways: dropwise, all at once (one-shot), and using a specially designed microfluidic device (Fig. 6). Next, a rotary evaporator removed the organic solvent from the nanoparticles. The detailed formulation is described below.

We used PCL as the primary material, THF as a solvent, water as a non-solvent, and F127 as a surfactant to limit the formulation area. For all tests, we used a constant magnetic stirring of 1000 rpm. Each formulation was performed in triplicate, and each measurement was repeated three times to eliminate outliers and validate the experimental determination of particle size within the same formulation.

### Classic nanoprecipitation

For the classic nanoprecipitation method, the organic phase was added dropwise via the syringe pump (LEGATO 210; KD Scientific Inc., Holliston, MA, USA) at a constant dropping rate (0.15 mL/min) to the beaker containing the aqueous phase with surfactant under magnetic stirring at room temperature. The obtained formulation was stirred magnetically for 10 min. The organic solvent was subsequently evaporated under reduced pressure using a rotary evaporator.

### One-shot method

For this method, the beaker containing the aqueous phase was placed on a magnetic stirrer, and the organic phase was transferred into a 6 ml syringe and rapidly injected into the stirring aqueous phase. Then, the formulation was stirred magnetically for 10 min. The organic solvent was subsequently evaporated under reduced pressure using a rotary evaporator.

### Microfluidic device

The microfluidic device was designed and 3D-printed as described previously^56^ (see Supplementary Figure S1 for the device layout and dimensions). Briefly, the microfluidic device design was created using Blender 3.0 software and 3D-printed using a ZMorph VX printer (ZMorph, Wrocław, Poland). The printed model was coated with PDMS resin and cured at 90 °C for 15 min. After removing the 3D-printed model, a partially cured PDMS flat piece was placed over the hollow space, followed by a 1 kg weight. The device was left to cure overnight at 90 °C. Silicone tubing with an inner diameter of 3 mm was inserted into the PDMS chip inlets and outlets and sealed with silicone glue.

For the NP formulation using the microfluidic device, the syringes containing organic and aqueous phases were placed in the syringe pumps (LEGATO 210; Ascor AP-14, Ascor Med, Sp. z o.o., Warsaw, Poland, respectively) and connected to the module. The flow rate ratio^56,57^ of the aqueous phase to the organic phase was 200:1, and the precipitation process was carried out at room temperature. After completion, the suspension was stirred magnetically for 10 min. The organic solvent was subsequently evaporated under reduced pressure using a rotary evaporator.

Microfluidic devices were used to precisely control mixing conditions during nanoprecipitation. The laminar flow regime in the microchannels, characterized by low Reynolds numbers (Re < 2000), ensured well-defined interfaces between the organic and aqueous phases. This controlled environment minimized turbulence, allowing diffusion-driven mixing and uniform nanoparticle formation. The flow rate ratio of aqueous to organic phase was set at 200:1 to achieve hydrodynamic focusing, where the solvent diffusion into the antisolvent triggered nanoprecipitation.

### Effect of PCL concentration on droplet size and nanoparticle formation

The organic-phase viscosity was adjusted by modifying the polymer concentration (1–370 g/L). For one-shot nanoprecipitation, EtOH was added to THF as a co-solvent (EtOH: THF = 1:2, v/v) to improve protocol robustness across the investigated concentration range. For the classical dropwise experiments, the organic phase was THF without ethanol unless stated otherwise.

### Surfactant adsorption and stability

F127 was used as a non-ionic surfactant and dissolved in ultrapure water at concentrations ranging from 0.10 to 6.35 g/L. The aqueous surfactant solution served as the anti-solvent phase in which nanoprecipitation occurred. The objective was to examine the effect of surfactant concentration on nanoparticle size, colloidal stability, and polydispersity.

### Particle size analysis

Dynamic Light Scattering (DLS) was performed using a Malvern Zetasizer Nano ZS (Malvern Instruments, Malvern, UK) at a scattering angle of 173° and at room temperature (25 ± 0.5 °C). All samples were measured in triplicate at a pH of 7.4. The final colloidal concentration for DLS measurements was adjusted to avoid multiple scattering and maintain an appropriate photon count rate. Throughout the manuscript, the mean hydrodynamic diameter is reported as number-weighted (D_h_), obtained by converting intensity-weighted distributions to number-weighted using the instrument’s Mie-theory model with the specified optical constants. The polydispersity index (PDI) is derived from the cumulant analysis of the intensity autocorrelation function. The size distributions obtained were unimodal. The surface charge measurements characterized by the zeta potential (ζ) were conducted in ultrapure water with 0.1 M PBS buffer (pH 7.4) in a 9:1 volume ratio. All measurements were performed in triplicate to ensure reproducibility.

### Solution rheology

The rheological properties of the polymer solutions were measured using a rotational rheometer (MCR102e, Anton Paar GmbH, Austria) equipped with an air-bearing-supported EC motor and a cone-and-plate geometry (CP50-1 cone diameter, 50 mm; angle, 1°; gap, 0.05 mm). Before all measurements, polymer solutions were allowed to equilibrate for 24 h at 20 °C to ensure complete polymer hydration and removal of dissolved air bubbles through gentle vacuum degassing. Steady-state shear viscosity as a function of shear rate was determined over the range 0.1–100 s^− 1^ using a controlled-shear-rate protocol. The temperature was maintained at 20 ± 0.1 °C using a Peltier heating system. Before each measurement, a pre-shear step was applied (shear rate 100 s^− 1^ for 60 s) followed by a 5-minute rest period to minimize the influence of shear history on the measured viscosity. Both increasing and decreasing shear rate ramps were recorded to assess potential thixotropic behavior. All experimental data were collected and analyzed using RheoCompass software with applicable measurement templates. Results were exported and further processed using Origin Pro 2021 (OriginLab Corporation, USA) for visualization.

## Supplementary Information

Below is the link to the electronic supplementary material.

Supplementary Material 1

## Data Availability

The data presented in this study are available on request from the corresponding author.
