# Supplementary material for: Modeling and experimental verification of polycaprolactone nanoparticle precipitation
Source: Sci Rep. 2026 Jan 29;16:6613. doi: 10.1038/s41598-026-35286-y (PMC12913609; doi:10.1038/s41598-026-35286-y)
Supplement: Supplementary file 1 — Supplementary Material 1 [file 41598_2026_35286_MOESM1_ESM.docx]

**Supplementary information for “Modeling and Experimental Verification of Polycaprolactone Nanoparticle Precipitation”**

**Ewa Rybak ^1,2*^, Jakub Trzciński ^2,*^, Jakub Gac ^1^, Tomasz Ciach ^1^**

^1^ Faculty of Chemical and Process Engineering, Warsaw University of Technology, Waryńskiego 1, 00-645 Warsaw, Poland; ewa.rybak@pw.edu.pl (E.R.); jakub.gac@pw.edu.pl (J.G.), tomasz.ciach@pw.edu.pl (T.C.)

^2^ Centre for Advanced Materials and Technologies CEZAMAT, Warsaw University of Technology, Poleczki 19, 02-822 Warsaw, Poland; Phone: +48 22 182 11 94; Fax: +48 22 621 6892; ewa.rybak@pw.edu.pl (E.R.); jakub.trzcinski@pw.edu.pl (J.T.)

* Correspondence: [ewa.rybak@pw.edu.pl](mailto:ewa.rybak@pw.edu.pl); [jakub.trzcinski@pw.edu.pl](mailto:jakub.trzcinski@pw.edu.pl)


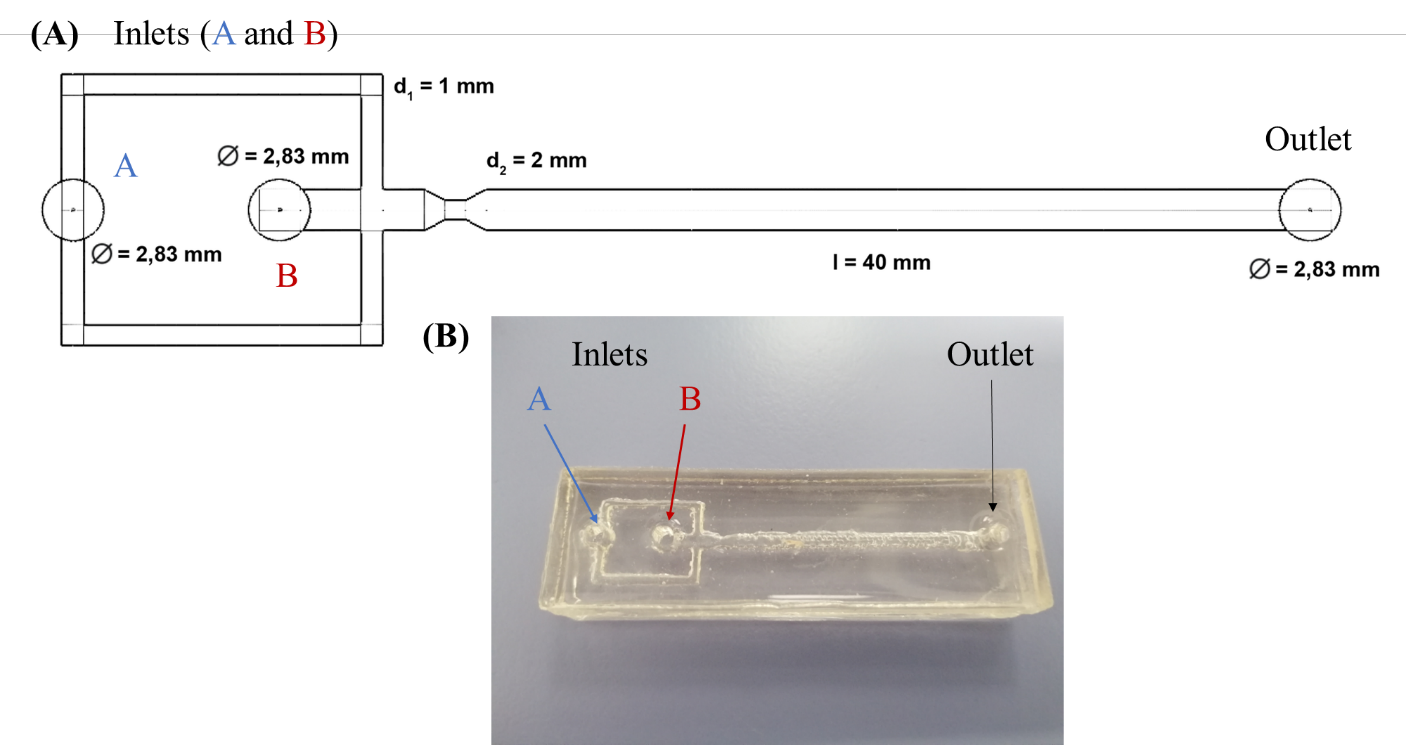


**Supplementary Figure S1.** (A) Schematic of the microfluidic device. (B) Flow-focusing microfluidic device for NP formulation. Inlet A (blue) - aqueous phase, inlet B (red) - organic phase.

**Supplementary Note 1: Influence of formulation method on nanoparticle size and PDI**

All samples described in this section were prepared with a fixed surfactant concentration of 6.35 g/L. The hydrodynamic diameter (D_h_) as a function of PCL concentration showed similar trends for all three formulation methods—dropwise, one-shot, and microfluidic (Supplementary Figure S2). To quantify these observations, a two-way ANOVA with formulation method and PCL concentration as fixed factors was performed. For D_h_, the main effect of formulation method was not statistically significant (p > 0.05), whereas PCL concentration had a significant effect (p = 0.0011), confirming that nanoparticle size increases systematically with polymer concentration irrespective of the mixing strategy. For PDI, neither the formulation method nor PCL concentration showed a statistically significant main effect (p > 0.05). These results indicate that, under the optimized conditions used in this work, the three mixing strategies yield comparable nanoparticle sizes and polydispersity, and that polymer concentration is the dominant parameter governing D_h_ within the investigated range of formulation conditions.

**
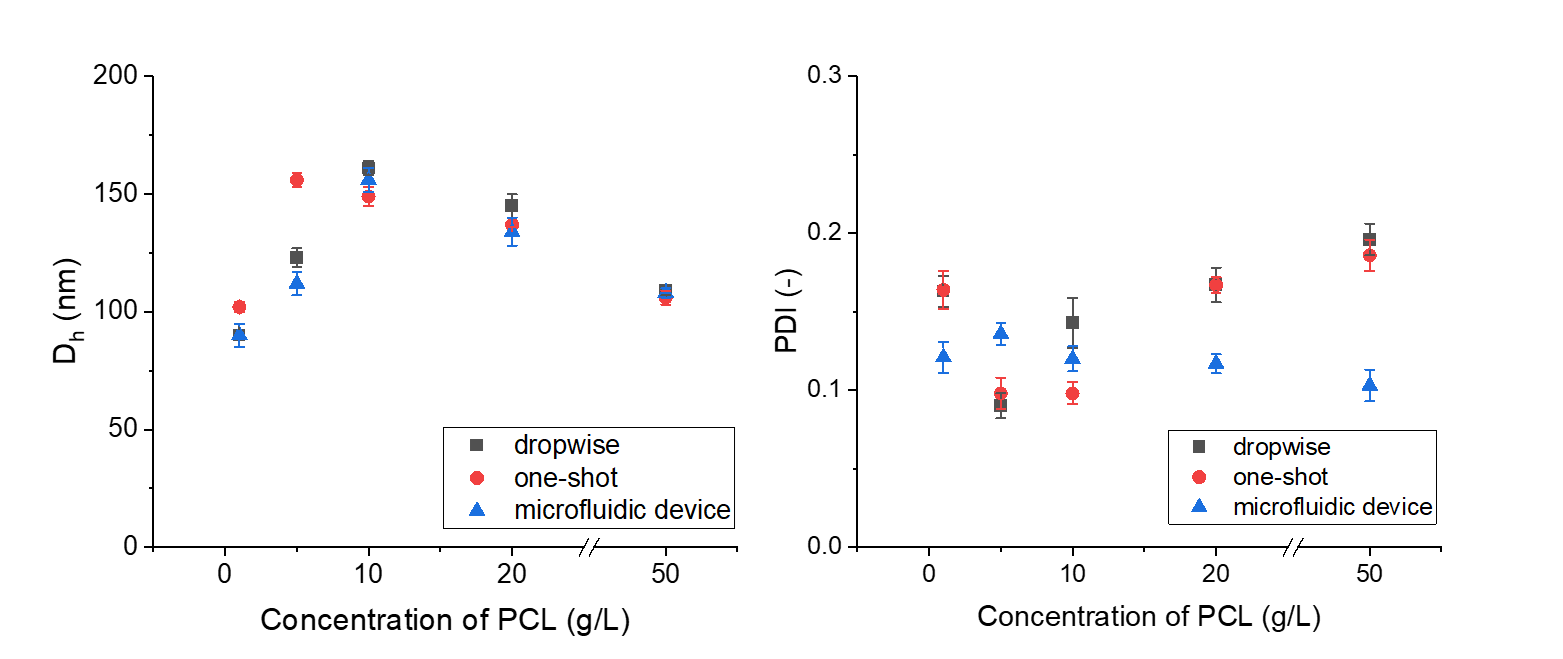
**

**Supplementary Figure S2.** DLS measurements of PCL nanoparticle suspensions formulated using the dropwise, one-shot, and microfluidic methods at different PCL concentrations (6.35 g/L F127, n = 3). Data are shown as mean ± SD. Two-way ANOVA (factors: formulation method and PCL concentration) showed no statistically significant differences in D_h_ or PDI between the three methods (ns, p > 0.05 for the main effect of formulation method). In contrast, PCL concentration had a significant effect on D_h_ (p = 0.0011), whereas its effect on PDI was not significant (p > 0.05), indicating that under the conditions investigated, nanoparticle size, but not polydispersity, exhibits a statistically significant dependence on polymer concentration.

**Supplementary Table S1. DLS measurements of selected NPs obtained with the one-shot method, with the addition of EtOH in a ratio of 1:1 and 1:2 (THF/EtOH). NP suspensions without surfactant addition, with a constant PCL concentration. Data shown as mean ± SD (standard deviation).**

| Volume ratio | | Volume [ml] | | | D_h_ ± SD [nm] | PDI ± SD [-] | *ζ* potential [mV] |
| --- | --- | --- | --- | --- | --- | --- | --- |
| THF | EtOH | THF | EtOH | H_2_O |  |  |  |
| 1 | 2 | 4 | 8 | 48 | 132 ± 2 | 0.043 ± 0.008 | -10 |
|  |  |  |  | 96 | 63 ± 1 | 0.084 ± 0.012 | -11 |
|  |  |  |  | 48 | 107 ± 5 | 0.034 ± 0.009 | -12 |
|  |  | 10 | 20 | 220 | 197 ± 4 | 0.114 ± 0.018 | -8 |
| 1 | 1 | 4 | 4 | 48 | 123 ± 3 | 0.049 ± 0.003 | -10 |
|  |  |  |  | 96 | 104 ± 3 | 0.065 ± 0.012 | -11 |
|  |  |  |  | 48 | 142 ± 3 | 0.094 ± 0.013 | -12 |
|  |  | 10 | 10 | 220 | 208 ± 7 | 0.078 ± 0.007 | -9 |

**Supplementary Table S2. DLS measurements of selected NPs obtained with the one-shot method. with the addition of EtOH in a 2:1 ratio (THF/EtOH). NP suspensions without surfactant addition. PCL concentration constant. Data shown as mean ± SD (standard deviation).**

| Volume ratio | | Volume [ml] | | | D_h_ ± SD [nm] | PDI ± SD [-] | *ζ* potential [mV] |
| --- | --- | --- | --- | --- | --- | --- | --- |
| THF | EtOH | THF | EtOH | H_2_O |  |  |  |
| 2 | 1 | 4 | 2 | 48 | 127 ± 1 | 0.059 ± 0.005 | -14 |
|  |  | 8 | 4 | 96 | 132 ± 7 | 0.063 ± 0.007 | -15 |
|  |  | 12 | 6 | 144 | 161 ± 8 | 0.056 ± 0.005 | -11 |

**Supplementary Table S3. Summary of formulation and process parameters affecting PCL nanoparticle size and distribution.**

| **Parameter** | **Effect on NP size** | **Effect on PDI** | **Mechanism** |
| --- | --- | --- | --- |
| **Polymer concentration (PCL)** | Increase → larger NPs | Increase → higher PDI | Higher viscosity of the organic phase promotes droplet growth over nucleation. |
| **Surfactant presence (F127, 0.5% w/v)** | Addition → slightly larger NPs | Addition → lower PDI (more uniform) | Steric stabilization by F127 limits aggregation and promotes homogeneous populations. |
| **Surfactant concentration** | Increase → smaller NPs; diminishing returns at high levels | Slight increase in PDI at higher concentrations | More interfacial coverage slows coalescence; adsorption saturates, and micellization reduces free surfactant |
| **Co-solvent (EtOH, 1:2 EtOH: THF)** | At low PCL conc.: smaller NPs; at higher PCL conc.: may promote extended growth (larger NPs) | Improves robustness/reproducibility at the optimized ratio; PDI is mainly governed by PCL concentration | EtOH improves miscibility and solvent diffusion; under some conditions, slower precipitation can extend growth. |
| **Mixing method (dropwise, one-shot, microfluidic)** | Comparable mean sizes under optimized conditions | No significant main effect under optimized conditions (microfluidics may reduce variability) | Microfluidics ensures rapid, diffusion-driven mixing; dropwise and one-shot are more variable. |
